# Safety analysis over time: seven major changes to adverse event investigation

**DOI:** 10.1186/s13012-017-0695-4

**Published:** 2017-12-28

**Authors:** Charles Vincent, Jane Carthey, Carl Macrae, Rene Amalberti

**Affiliations:** 1 Department of Experimental Psychology, University of Oxford, 15 Parks Road, Oxford, OX1 3PW UK; 2 Jane Carthey Consulting, London, UK; 3 Haute Autorité de Santé, Paris, 5 Avenue du Stade de France, Saint-Denis, 93210 Paris, France

**Keywords:** Patient safety, Incident analysis, Safety interventions

## Abstract

**Background:**

Every safety-critical industry devotes considerable time and resource to investigating and analysing accidents, incidents and near misses. The systematic analysis of incidents has greatly expanded our understanding of both the causes and prevention of harm. These methods have been widely employed in healthcare over the last 20 years but are now subject to critique and reassessment. In this paper, we reconsider the purpose and value of incident analysis and methods appropriate to the healthcare of today.

**Main text:**

The primary need for a revised vision of incident analysis is that healthcare itself is changing dramatically. People are living longer, often with multiple co-morbidities which are managed over very long timescales. Our vision of safety analysis needs to expand concomitantly to embrace much longer timescales. Rather than think only in terms of the prevention of specific incidents, we need to consider the balance of benefit, harm and risks over long time periods encompassing the social and psychological impact of healthcare as well as physical effects.

We argued for major changes in our approach to the analysis of safety events: assume that patients and families will be partners in investigation and where possible engage them fully from the beginning, examine much longer time periods and assess contributory factors at different time points in the patient journey, be more proportionate and strategic in analysing safety issues, seek to understand success and recovery as well as failure, consider the workability of clinical processes as well as deviations from them and develop a much more structured and wide-ranging approach to recommendations.

**Conclusions:**

Previous methods of incident analysis were simply adopted and disseminated with little research into the concepts, methods, reliability and outcomes of such analyses. There is a need for significant research and investment in the development of new methods. These changes are profound and will require major adjustments in both practical and cultural terms and research to explore and evaluate the most effective approaches.

## Background

Every safety-critical industry devotes considerable time and resource to investigating and analysing accidents, incidents and near misses. Such industries employ many methods for assessing safety. Incident reporting and investigation, often called ‘lagging indicators’ are complemented by what are termed ‘leading indicators’, such as human reliability analysis, safety audits and horizon-scanning for future migrations from safety procedures. While lagging indicators focus on learning from failure and non-compliance, leading indicators support organisations to anticipate future safety risks. There is a rich literature outlining the benefits and limitations of specific types of lagging and leading indicators, and it is generally accepted that how well an organisation combines learning from the two types of indicators optimises safety performance [1–3]. Analyses of safety issues in any industry therefore requires review of a range of information [4, 5]; nevertheless, the identification and analysis of serious incidents and adverse events continues to be a critical stimulus and guide for safety improvement.

Implementation science has primarily focussed on improving the uptake of existing research evidence, interventions and policy but may also address the development of interventions. The findings of incident analysis can be valuable here in identifying vulnerabilities in systems and suggesting interventions that may improve safety and reliability. This may occur through the analysis of single incidents within an organisation or through aggregated analyses of incidents at a national level. For instance, Rees et al. [6] recently reviewed 2191 paediatric safety incidents, including 12 deaths and 41 cases of severe harm. In their examination review of potential solutions to the problems identified, they prioritised medication provision in community pharmacies; triage processes to enable effective and timely assessment, diagnosis and referral of acutely sick children attending out-of-hours services; and enhanced communication for robust safety netting between professionals and parents [6]. The analysis of incidents can therefore act as a powerful stimulus and guide to the development and implementation of interventions to improve healthcare.

A critical challenge for patient safety in earlier years was to develop a more thoughtful approach to both error and harm to patients [7, 8]. While a particular action or omission may be the immediate cause of an incident, closer analysis usually reveals a series of events and departures from safe practice, each influenced by the working environment and the wider organizational context [9, 10]. We previously extended Reason’s organisational accident model and adapted it for use in healthcare, classifying the error producing conditions and organizational factors in a single broad framework of factors affecting clinical practice [7]. This gave rise to a method of incident analysis often referred to as ALARM [11] and a later revision and extension in 2004 [12] (the ‘London Protocol’) which has been translated into several languages and can be applied to all areas of healthcare including the acute sector, mental health, and primary care. Similar methods, usually referred to as ‘root cause analysis’, have been developed and implemented by a number of organisations [8, 13]. In this paper, we have chosen not to use the term root cause analysis as, like other commentators, we believe that the ideal of a single root cause is thoroughly misleading [14, 15].

The systematic analysis of incidents has greatly expanded our understanding of both the causes and prevention of harm. These analyses have been conducted in multiple clinical settings and have revealed both the range of vulnerabilities in health systems but also the many factors that may contribute to error [6, 16–19]. For instance, in a review of medication-related incidents [20], Dean and colleagues identified a wide variety of specific errors and contributory factors which in turn suggested methods of intervention and error reduction. These approaches have been widely employed in healthcare over the last 20 years but are now subject to critique and reassessment [5, 15, 21, 22]. We need to reconsider the purpose and value of incident analysis and develop methods appropriate to the healthcare of today.

### The need for reassessment

The primary need for a revised vision of incident analysis is that healthcare itself is changing dramatically [4]. People are living longer, often with multiple co-morbidities which are managed over very long timescales. Care is increasingly being delivered in the home and in community settings; patients and families are increasingly and necessarily in charge of their own care. The emphasis and indeed purpose of healthcare is changing from one of healing and restoration to health to the longer term aim of supporting well-being in the face of chronic disease and increasing frailty [23]. The focus of care is moving from the hospital to the home and community which means that we need to pay much more attention to safety issues that arise outside at home, in the community and in primary care settings.

We have recently argued [4] that our vision of safety analysis needs to expand concomitantly to embrace much longer timescales. The analysis of specific incidents remains important but there is increasing evidence that the harm and failures of care that patients may suffer are often due to an accumulation of problems over a long time period across multiple contexts [24, 25]. For instance, many hospital admissions for older people are due to adverse drug events, particularly those due to the interaction of multiple medications over a long period of time. Analyses of incidents generally limited to a single hospital admission or to a specific event in primary care but many adverse drug events can only be understood by examining decisions and events along the patient journey. In the case of an admission to hospital with a complex drug reaction, a full understanding of the event might only be achieved by tracking the history of the patient’s care over several months leading up to the admission. Patients’ and carers’ perspectives are rarely sought and yet they may be the only people who can provide a picture of the care delivered over long time periods [4].

The second major reason for a reassessment is that many of the analyses that are conducted do not lead to effective actions or improvements. Reviews conducted by healthcare organisations suggest that the implementation and quality of incident analysis can be highly variable and is often poor [5, 8, 15, 26]. Organisations are often under pressure to deliver a large number of mandated investigation reports which means that the analyses can degenerate into a bureaucratic process in which the only outcome is the production of a report identifying ‘root causes’ and making formulaic and largely unachievable recommendations. For instance, in a recent analysis of 302 incident analyses, Kellog and colleagues [27] found that the most common recommendations were for more training and the reinforcement of existing policies neither of which, even if implemented, were likely to have any substantial impact on the problems uncovered. They found that multiple event types were repeated in the study period in the same organisation, despite repeated analyses. This suggests that we have perhaps been too optimistic in assuming that a thoughtful analysis would lead naturally to reasonable and effective recommendations; rather, we need to give much more thought to how recommendations and interventions should be derived from incident analysis data and to draw on a richer set of recommendations and responses [4, 6].

## Main text

### Understanding the whole patient journey: an illustrative case

To assist our reflections on the future of safety analysis, it is helpful to reflect on an illustrative story of the death of a person with mental health problems from a cardiac arrest while being treated as an inpatient and after having been prescribed a new generation of anti-psychotic drugs (Table 1). The story demonstrates the valuable insights we can get from seeing events through the eyes of the patient and how the time frame for analysis needs to cover the patient’s journey from the point of them presenting with symptoms across multiple healthcare providers.

**Table 1:** A mental health service user dies of a cardiac arrest

A mental health service user died from a cardiac arrest whilst being treated as an inpatient after being admitted to an acute mental health ward. Patient B had been prescribed a new generation anti-psychotic drug used in the treatment of schizophrenia (clozapine). Review of patient B’s notes over a 4-year time frame identified several recorded entries where she had raised concerns about the cardiac side effects of clozapine. She had been experiencing heart palpitations and was diagnosed with tachycardia following referral for cardiac review. During this 4-year time frame, patient B was living independently in the community, supported by her family, a community mental health team and GP. She made repeated requests for her medication to be changed to the GP and community mental health team. Her concerns were not acted on even though the National Institute for Health and Care Excellence (NICE) guidelines on the prevention and management of schizophrenia emphasise that the choice of anti-psychotic medication should take the patient’s views into account: The United Kingdom NICE Guideline CG 178, Psychosis and Schizophrenia in Adults: prevention and Management states: ‘The choice of antipsychotic medication should be made by the service user and healthcare professional together, taking into account the views of the carer if the service user agrees. Provide information and discuss the likely benefits and possible side effects of each drug, including: • metabolic (including weight gain and diabetes) • extrapyramidal (including akathisia, dyskinesia and dystonia) • cardiovascular (including prolonging the QT interval) • hormonal (including increasing plasma prolactin) • other (including unpleasant subjective experiences).’ Because of the physical symptoms she was experiencing, patient B repeatedly stopped taking clozapine. The healthcare professionals involved in her care focused on persuading patient B she should keep taking clozapine. There is no evidence anyone considered ‘Patient B is experiencing physical side effects from taking clozapine. The heart palpitations she is experiencing are causing considerable anxiety. She is at high risk of medication non-compliance.’ She eventually died of a cardiac arrest while in hospital for her mental health problems.	

Supporting and treating patients with co-existing mental ill health and physical health symptoms can be very challenging, and it is perhaps easy with hindsight to say that this person’s own account of her cardiac symptoms should have been taken more seriously. It is clear however that the healthcare professionals involved became fixated on persuading patient B to take a medication she was reluctant to use. Over time, her feelings of anxiety about taking clozapine increased and she exhibited increasing paranoia, believing the healthcare team were encouraging her to take a medication that was harmful to her cardiac health. She was in fact entirely correct in her assessment.

The scope of the hospital investigation into patient B’s death focused on the period from the time of her acute inpatient admission to her death on the ward. The fact that she had been seen as a ‘non-complaint’ patient and the events leading up to her admission were hardly considered at all. The care given by the hospital in the last days of the patient’s life was in fact of good quality and shed little light on the reasons for her decline and untimely death. Had the time frame for analysis been extended to capture the last months and years of the patient’s healthcare journey and investigated how the drift away from treatment recommended in guidelines occurred, the findings from the analysis may have been very different. They would, for instance, have focused more on how using persuasion as a strategy to convince a mental health service user who is raising concerns about their prescribed medications leads to a breakdown in the therapeutic relationship between care provider and patient and prolonged psychological harm.

This illustrative story serves to introduce some of the changes needed in our approach to analysing the risks of healthcare. We clearly need to examine much longer time periods, but the changes needed are much more than an extension of the current methodology, even though many of the underlying concepts and methods can still provide a relevant foundation. In particular, we need to look at fewer incidents in much greater depth, to be led much more by patients, to seek to understand success and recovery as well as failure and finally to consider an expanded repertoire of responses and recommendations.

### Seven major changes to adverse event analysis

#### Widen the time frame of analysis: review the patient journey

In many cases, the time period encompassed by safety analysis will need to be extended to examine a significant portion of the patient’s journey through the healthcare system, exploring the safety of the patient journey through time. This is different to simply examining the causal history of factors that led to the occurrence of one particular event. Many patients suffer significant harm because of multiple small failures that accumulate throughout their care, rather than a single dramatic failure at one point in time [24, 28–30]. We already understand that after an incident we need to look back to the series of factors that combined to create a single harmful event—for instance, a young doctor is unsupervised at night with inadequate equipment, a difficult team and a very sick patient. More often though, in the care of a patient over time, we see a progressive degradation in care due to a combination of errors and system vulnerabilities that accumulate over a long period.

Amalberti and colleagues [31, 32] have previously argued that we should extend the time frame of analysis to consider an ‘event journey’. However, to examine safety over longer time periods, particularly in community settings, we now believe that we should speak simply of the patient journey. This will require looking back through the medical history of the patient in search for all events that have defined the patient’s journey and contributed to the final outcome, whether or not these events have been perceived as serious at the time they occurred or whether the problem was detected and resolved. In this scenario, we will need to try to understand, without the benefit of hindsight, both the benefits and risks of certain courses of action and how sometimes risks will be knowingly and acceptably taken because of the benefits incurred. A simple but important example is the frail person who elects to stay at home rather than be cared for in a residential setting; this course is medically more risky, but the risks are outweighed by the wider social and psychological benefits.

The suggestion that we might extend our analysis to longer time periods raises a number of questions about the definition and identification of events that would be suitable and productive for analysis. The word ‘incident’ does not seem entirely appropriate when considering a series of events that might gradually unfold over several months and some new terminology might be required. We might instead speak of a ‘safety analysis of the patient journey’ over a particular time period. We would also need to identify the trigger events which might lead to such analyses. In the first instance, these might be familiar adverse events which on initial inspection suggest a long genesis. In time, we might be able to identify unsafe periods of patient care, although this is not easy with current information systems.

#### Work with patients and families to identify, define and prioritise safety issues

Identifying periods of time when care has been unsafe is difficult with current systems firstly because they are not oriented to detecting problems of this kind and secondly because most healthcare staff will only be aware of particular episodes within the patient journey. One potential solution is to analyse some incidents that are selected by patients rather than by professionals to gain a wider coverage of the landscape of potential problems. Incidents currently selected for analysis by professionals tend to be those that have the most immediate and most visible impact in a particular clinical context. The longer term problems that patients can experience as they receive care across different clinical settings over long periods of time are invisible to most professionals not because they are unwilling to see them but simply because the information is not readily available [33, 34].

We already know that patients and families are able to reliably identify adverse events that have not been detected by professionals. Patients have been shown in a number of studies to report errors and adverse events accurately and to provide additional information not available to healthcare professionals [34–37]. We suggest that events considered for analysis should be selected from the patient’s point of view as well as by professionals. We do not yet know what other kind of events might be identified as worthy of investigation by patients and families but they may be quite different from those identified by professionals. Developing methods to identify significant events identified by patients will be challenging and will likely require collaboration between families and professionals to select events and thematic issues that provide the most fruitful insights [38].

The inclusion of patients and families in both selection and analysis is likely to profoundly affect both the focus and findings of safety analyses. We might anticipate for instance that much more attention will be given to psychological and social issues, that communication and the coordination of care between providers [34] will be a dominant theme and that the cognitive and emotional demands of caring will emerge as a safety issue in its own right.

#### Conduct fewer, deeper and more proportionate analyses

Healthcare organisations have finite resources for safety investigation and analysis, and these resources are frequently consumed by the demands of outside agencies. Staff can become understandably frustrated and burdened by requirements for time-consuming incident investigation carried out without adequate time or sufficient training. Much greater investment in training in safety analysis is certainly needed [15]. But more fundamentally, much more care and attention needs to be paid in selecting which incidents would be suitable for extensive analysis, particularly in terms of the likely improvements to patient safety. The depth and extent of the analysis conducted should ideally be proportionate to the value of the potential learning and improvements that are likely to result.

Some tragic events must be investigated because the patient and family need an explanation and support; this is right and necessary, and healthcare organisations have a moral responsibility to respond to families who have suffered [39–41]. However, these are not necessarily the events which provide the greatest insights into the strengths and vulnerabilities of the healthcare system. For the purposes of safety improvement, a fairly drastic ‘triage’ process should be carried out. Some issues can be reviewed fairly rapidly by a clinical team in a team meeting, while others with wider implications may require longer and more extensive analysis possibly coordinated  across multiple organisations [42]. At present, the main criteria for choosing incidents within organisations is the severity of outcome, although at national level, investigations may be prioritised because they are addressing issues that have widespread implications for the whole system of care [43, 44]. Severity of outcome could remain a criterion for longer term analyses, but we might also want to prioritise events that were likely to reveal problems not highlighted in current systems; these would certainly include problems that arose within the patient’s home, breakdowns in coordination of care across providers and difficulties in transitions between providers of care.

Such long-term analyses would be resource intensive. We suggest however that an extensive analysis of 10 incidents a year across clinical settings would provide just as rich an understanding of the landscape, richer than 100 rushed and formulaic analyses. A deep investigation of 10 incidents in a given clinical area over a year would reveal enough insight for several years’ worth of further exploration and improvement. Findings from such analyses must then be considered in the context of other safety relevant information [45] and the longer term organisational strategy for improving the quality and safety of care.

Applying a more proportionate approach to prioritising analytic resources also requires a flexible portfolio of analytic strategies. For example, productive analyses of minor issues can be carried out rapidly in a few minutes in a team meeting [39] or perhaps used as the basis for reflection in a larger clinical or organisational meeting. Where more detailed analysis may be required, escalating approaches such as concise analysis tools [42], a rapid SWARM analysis [46] through to system level reviews [47]. These all represent different approaches to reflection and investigation of safety issues at different levels across an organisation, which is foundational practice in other safety critical industries [43]. The critical point is to triage incidents and events and customise an appropriate degree of inquiry.

#### Understand success and failure in detection and recovery

Analyses and reports should identify, and indeed celebrate, good treatment as well as pointing out failures in the care process [48, 49]. More subtly, we also need to pay more attention to both successes and failures of detection, anticipation and recovery [4]. There is much to learn from the ability of the system to detect and recover from failures and close calls [38, 50]. For example, in addition to identifying failures and contributory factors, we could instead ask ‘what successes and failures of response and recovery occurred in the care of this patient?’ and ‘how we can we improve detection and recovery in settings such as these?’ These questions are fundamental to understanding how safety functions and risk controls work effectively—and how they break down [9]. They are also central to many prospective risk analysis methods, such as Bow Tie analysis [51] and Failure Mode and Effects Analysis [52]. Bow Tie analysis, for instance, has been extensively used in the aviation sector to identify and communicate risk information effectively. Carrying out a prospective analysis of a safety risk using the Bow Tie method involves asking a structured set of questions prospectively addressing the nature of a hazard, the circumstances that lead to danger, the impact of loss of control and the means of recovery and mitigation of any harms. Bow Tie diagrams illustrate a hazard, the undesirable event, trigger events (threats) and potential outcomes; they also show the risk controls put in place to minimise the risk. Failure Modes and Effects Analysis, occasionally used in healthcare, [53–55] is another analytical method that provides a systematic and structured approach to identifying potential sources and consequences of failure. A key stage of this method involves identifying how potential failures might be detected and responded to if they actually occur.

We should also give serious consideration to examining events and episodes where a successful recovery occurred which would be supportive of staff morale and also instructive in revealing successful adaptive strategies, such as ‘good catch’ programmes in healthcare that seek to analyse and understand events where staff effectively detected and recovered from potentially harmful events [56]. This would have implications both for our understanding of events and, more importantly, for the recommendations which follow such analyses which might expand to include a much stronger focus on developing detection and recovery strategies.

#### Examine safety issues and contributory factors at different time points

The original ALARM/LONDON protocol proposed that, after the initial care delivery problems were identified, each should be analysed separately to consider the contributory factors [11]. In a sequence of problems, different sets of contributory factors may be associated with each specific problem. For instance, a nurse might fail to ask for advice about a deteriorating patient due to inexperience, poor supervision and deficiencies in teamwork; in contrast, the same patient might later fail to receive the correct medication, but this might be due to inadequate staffing and poor organisation of care. With an analysis over a relatively short timescale, this more complex analysis may not be carried out and all the contributory factors may be considered together; in fact, techniques such as the fish bone causal diagram actively promote the aggregation of all contributory factors into a single amalgam. Much of the value of mapping out the complex patterns of factors and conditions that underlie safety issues is therefore lost, as fine-grained explanations of how events evolve and interlink can instead give way to simplistic lists [57]. However, this more subtle perspective becomes much more important with a longer timescale as a series of problems may be identified which are clearly separated in time and context. Each of these can be separately analysed using the ALARM grid to build up a much more detailed picture of system vulnerabilities.

#### Reflect on the workability of the underlying care process

Any safety analysis begins by comparing what actually happened with what ideally should have happened. Indeed, it is typically a dramatic mismatch between what was expected and what actually happened that alerts organisations to a serious safety problem in the first place [58]. Analysing incidents in terms of the gap between the way work is expected to be done and what actually occurs is a central strategy in detecting the early signs of emerging safety problems [59–61]. Comparing how work is actually done and how it is imagined is one of the primary learning opportunities offered by incident analysis in other industries [43]. For example, in airline incident reporting and investigation systems, safety investigators assume that knowledge of organisational practice is always partial and imperfect and so focus considerable attention on identifying any discrepancies between the practices that are described in incident reports and those that are specified in formal manuals and standard procedures. Even minor discrepancies, such as the order in which bolts are routinely replaced in a maintenance task, can cause serious concern. These discrepancies will typically prompt a detailed investigation of the nature of the ‘real-world’ practical work involved, along with careful examination of the formal procedures that are intended to guide this work, to ensure that both are aligned, reliable and mutually supportive [43, 62].

Analysing this mismatch allows broader reflection on the nature of the problems identified and wider system vulnerabilities. However, typically, the accepted standard of care is taken as a given and the analysis examines reasons for departure from that standard. The conclusion of the investigation may simply be that staff did not comply with standard procedures without any consideration of the workability of those procedures [63] or the context and circumstances in which the care was delivered. Adherence to processes and standards is rightly ingrained in the professional training of nurses and other professions which may make such reflections appear non-professional. Investigations therefore need to look more closely not only at the reasons for departures from standard procedures but at the standards and procedures themselves [64]. This is a subtle but important change of analytic stance. When analytical efforts are directed at identifying and understanding the mismatch between expected organisational work and the real situated practices of professionals, it simultaneously allows both to be updated, improved and revised. There is potential for using this perspective more actively in longer term reviews of safety issues to highlight the workability or unworkability of the intended care processes and so to develop smarter ways of working that are more aligned to human capacities.

#### Broaden our repertoire of responses and recommendations

Investigations have to be completed in a timely manner and the immediate priority is often to provide assurance to hospital patient safety committees, commissioners and regulators that action plans have been generated and recommendations have been implemented. In their turn, committees and regulators seek assurance that the plan has been actioned in a timely manner, an approach which is guaranteed to encourage simple recommendations that avoid thorny system problems. Reviews of recommendations generated by incident analysis have found that most recommendations are weak and few address fundamental system issues [5, 21, 44]. There is little understanding that people-focused solutions [65, 66] like adding in another double-check, re-writing the safety procedure, creating a new safety procedure or sending out an email reminder to staff are weak and will not in themselves lead to sustained improvements. Studies of successful improvement and behaviour change suggest that even when basic procedures are very well understood considerable work is needed both to understand reasons for non-compliance and to bring about change. Such changes, in the case of hand hygiene for instance, often require long term multi-faceted interventions which embrace cognitive, social and emotional determinants of behaviour all set within a wider organisational strategy with highly visible leadership commitment [67–70].

Analysts use frameworks of contributory factors to guide the investigation of incidents and we may need to also use frameworks of solutions and interventions to ensure that all potentially relevant interventions are considered [71]. We have previously distinguished five broad strategies each associated with a family of interventions [4]. The first two strategies aim to optimise care, improving both safety and efficiency; the first strategy is to target specific harms or specific clinical processes and the second aims at the improvement of the underlying work systems and processes. These two broad strategies are complemented by strategies that are more concerned with detecting and responding to risk and which assume, particularly in a time of rising demand and financial austerity, that care will often be delivered in difficult working conditions. These three additional strategies are risk control; monitoring, adaptation and response; and mitigation. Each of these is associated with a family of specific interventions. Reports after incident analyses typically consider only a very few of the dozens of potential strategies and interventions available. The development of frameworks of contributory factors 20 years ago [7] helped us structure investigation and ensure a comprehensive exploration of relevant issues; we now need to draw on a menu of safety interventions which we can review when considering what actions might be required following analyses of patient journeys.

## Conclusions

Healthcare is changing rapidly with more care being provided in the home and community to an ageing population suffering chronic diseases over long time periods. Our previous concepts of quality and safety have to evolve to reflect these changes. Rather than thinking primarily in terms of specific incidents, we need to consider the balance of benefit and harm over long time periods and encompass the social and psychological impact of healthcare as well as physical effects. We have argued that we need to look at the evolution of patient journeys in much greater depth, be more proportionate and strategic in analysing safety issues, assume that patients and families will be partners in investigation, seek to understand success and recovery as well as failure and develop a much broader and more thoughtful approach to recommendations (Table 2). The longer term perspective has important implications for the subsequent safety strategies that we implement. These changes are profound and will require major adjustments in both practical and cultural terms and research to explore and evaluate the most effective approaches.

**Table 2:** Seven major changes in adverse event analysis

Change in practice	Current	Future
Select some events identified by patients and families	Decision to investigate determined by organisational and regulatory priorities	Select some events with longer term chronologies identified by patients and families Ask patients to tell their story of the episode of care, focusing both on what went well and poorly.
Widen the time frame of analysis: review the patient journey	Determine recent accident chronology	Widen the timeframe to the whole patient journey
Fewer, deeper analyses	Give equal attention to all serious incidents	Prioritise events which must be explained to patients and families, thereafter, triage events to identify those with maximum potential for system-wide learning
Success and failure in detection and recovery	Identify problems in process of care and contributory factors	Identify benefits of care as well as problems, and include detection and recovery from problems
Examining safety issues and contributory factors at different time points	Identify contributory factors	Identify contributory factors to each individual problem and to detection and recovery
Reflecting on the workability of the underlying care process	Assume the current standard of care as a given	Reflect on the feasibility and workability of current standards and practices and whether these need to be adjusted
Broadening our repertoire of responses and recommendations	Recommendations and developing an action plan	Select from the full portfolio of strategies and interventions

First and foremost, the involvement of patients and families will need to become the default option in an investigation. This has already been mandated by the Dutch Safety Inspectorate, with the important proviso that healthcare organisations do not have to involve patients and families where there is good reason not to do so [38]. The routine inclusion of patients and families in inquiries should increase the attention given to emotional and social aspects of care and give more emphasis to communication and coordination of care over time and across organisations. We will need to develop new approaches to analyses which allow the inclusion of patient perspectives without burdening families unduly.

Second, everyone involved in investigation needs to reflect on what proportion of the current investigative activity is really meaningful and how information gained from incident analysis should be linked to system improvement. The effectiveness of investigations needs to be evaluated in terms of the quality of the analysis and the depth and seriousness of the ultimate recommendations rather than simply in terms of meeting deadlines and the signing off action plans. Both regulators and healthcare organisations also need to realise that generating recommendations on the basis of a single incident may not be optimal. Aggregate analyses of safety investigations over a year, integrated with other related safety and quality data, will allow examination of major system issues and the production of more meaningful action plans [6, 8, 72, 73]. For instance, poorly organised handover may be a factor in multiple safety incidents but be regarded as too difficult to tackle in a single incident action plan. A hospital reviewing a year’s worth of events however can see a pattern emerging and prepare a serious plan for improving handover across the whole organisation.

Third, safety analyses are commonly limited by the boundaries of the organisations in which the ultimate harm occurred. Analysing the patient journey, and seeing safety through the eyes of the patient, will necessitate the development of more sophisticated ways of conducting safety analyses across organisations including the home and community environment. Such activities will likely require the building of new investigative infrastructures, along with the development of norms and social agreements that support data sharing, collaborative analysis and coordinated improvement across organisational boundaries. This broader approach will require a new type of forum, supported by technical tools such as video conferencing, covering longer periods in the patient’s medical history and involving the participation of both hospital and community practitioners jointly with relatives and the patient. It would also require the development of new indicators and electronic traces, such as tools to monitor individual patients’ laboratory results, to record the nature and duration of all breakdowns in the continuum of care.

Fourth, implementing some of the changes we propose also requires us to improve the evidence available to those who lead safety analyses. Compared with other industries, healthcare investigations use a limited evidence base mainly consisting of the medical record and unstructured interviews [43, 74, 75]. The limited scope of information and evidence is a major contributor to the limited nature of many investigations and the lack of attention to system issues. All too often, current investigations are superficial because the quality of the evidence available to those leading the analysis is fragmented across different IT systems used by multiple providers or is of poor quality; for example, the scenario in which a medication error occurs and the infusion device memory has a 4-h capacity and an automatic over-ride built in, so the actual dose administered to a patient is never known because the device’s memory has been over-written by the time the investigation commences. With the introduction of new technologies and information systems, there are considerable opportunities to support the capture, retention and analysis of safety-relevant data [76, 77].

We recognise that the challenges of analysing even a small proportion of safety issues over longer time periods are considerable. Even with active patient and family involvement, it will be hard to track all relevant clinical information and to get an accurate sequence of events and contributory factors. In the British NHS and other national systems, the patient is at least notionally receiving care from a single provider although in practice the care is often provided by a mixture of publicly funded healthcare, social services and private providers. The challenges will be greater still in more fragmented systems such as the USA where the patient may receive care from a number of disparate and often unconnected healthcare providers. Even within those integrated systems in the USA, such as the Department of Veterans Affairs (VA) healthcare system, patients can receive care delivered or managed by the VA but also care from other providers for which VA has no regulatory authority. In many cases, it would not be clear who might initiate such safety analyses or be accountable for any identified system improvements. We would suggest however that it is nevertheless critical to attempt these analyses across multiple providers because this is the reality that patients and families face as they navigate these complex systems. We believe that many major safety issues are currently neglected precisely because they concern the coordination and integration of care across multiple providers.

Finally, there is a need for significant research and investment in the development of both current and new approaches. While the methods of incident analysis proposed 20 years ago have spread widely, there has been distressingly little research into the concepts, methods, reliability and outcomes of such analyses. We need, for instance, to develop and evaluate means of involving patients and families, to develop methods of assessing and monitoring the quality of investigations and to consider how the reliability and validity of the findings and recommendations from investigations might be meaningfully assessed. This period of revision offers an opportunity for a more critical and scientific vision to emerge which is truly linked to organisation and system-wide learning and improvement.
